# A simplified model to estimate nonlinear turbulent transport by linear dynamics in plasma turbulence

**DOI:** 10.1038/s41598-023-29168-w

**Published:** 2023-03-16

**Authors:** Tomonari Nakayama, Motoki Nakata, Mitsuru Honda, Emi Narita, Masanori Nunami, Seikichi Matsuoka

**Affiliations:** 1 The Graduate University for Advanced Studies, SOKENDAI, Toki, Gifu 509-5292 Japan; 2 National Institute for Fusion Science, Toki, Gifu 509-5292 Japan; 3 PRESTO, Japan Science and Technology Agency, 418, Honcho, Kawaguchishi, Saitama 332-0012 Japan; 4 Graduate School of Engineering, Kyoto University, Nishikyo, Kyoto, 615-8530 Japan; 5 National Institutes for Quantum Science and Technology, Naka, Ibaraki 311-0193 Japan; 6 Nagoya University, Graduate School of Science, Nagoya, Aichi 464-8603 Japan

**Keywords:** Magnetically confined plasmas, Nuclear fusion and fission

## Abstract

A simplified model to estimate nonlinear turbulent transport only by linear calculations is proposed, where the turbulent heat diffusivity in tokamak ion temperature gradient(ITG) driven turbulence is reproduced for a wide parameter range including near- and far-marginal ITG stability. The optimal nonlinear functional relation(NFR) between the turbulent diffusivity, the turbulence intensity $\mathcal{T}$, and the zonal-flow intensity $\mathcal{Z}$ is determined by means of mathematical optimization methods. Then, an extended modeling for $\mathcal{T}$ and $\mathcal{Z}$ to incorporate the turbulence suppression effects and the temperature gradient dependence is carried out. The simplified transport model is expressed as a modified nonlinear function composed of the linear growth rate and the linear zonal-flow decay time. Good accuracy and wide applicability of the model are demonstrated, where the regression error of $\sigma _{\textrm{model}} = 0.157$ indicates improvement by a factor of about 1/4 in comparison to that in the previous works.

## Introduction

Microscopic turbulence and related mass, momentum, and energy transport, which determines the confinement performance, are central issues in research of magnetically conned fusion plasmas. For the quantitative prediction of the turbulent transport fluxes caused by various microinstabilities such as the ion temperature gradient(ITG) driven mode, many efforts have been devoted to the rst-principle-based nonlinear gyrokinetic simulations^1^. In addition to recent advances in electromagnetic multi-species and multi-scale fluxtube simulations^2–5^, several validation studies with the experimental data have been carried out^6–8^. Furthermore, global turbulence simulations including external heat source and sink have revealed dynamic and non-local nature of the turbulent transport processes^9–14^. On the other hand, the gyrokinetic simulation often requires huge computational costs to investigate the long-time evolutions of the density and temperature profiles over the confinement time. The comprehensive numerical scans for various operation scenarios are, thus, still limited.

As another powerful approach to predict the long-time profile evolution and the power-balanced steady state, integrated transport codes, such as TASK/TASK3D^15–17^, TOPICS^18,19^, and GOTRESS+^20–22^, have been developed, where various simplified models based on experimental, theoretical, and numerical studies are combined. They individually calculate the magnetic equilibria, heating, fueling, neoclassical and turbulent transport, and time evolution of the macroscopic radial pressure prole. Particularly, turbulent transport models estimating the turbulent fluxes, which often exceed the neoclassical ones, have an important role in the prediction capability of the integrated simulation. There are many earlier works on constructing the simplified turbulent transport models in a wide variety of approaches, e.g., quasi-linear gyrokinetic and gyrofluid modelings^23–27^. Moreover, the effects of multi-scale fluctuations^28^, mean $\varvec{E}\times \varvec{B}$ flow shear^29^, and turbulence nonlinearity^30–34^ were investigated. Cross–field fluxes in the edge and scrape–off layer (SOL) region^35,36^ had also been modeled. Although the local transport approximation in terms of the turbulent diffusivity is often discussed, the importance of non–local and non-diffusive nature in a global turbulence system has also been revealed in several earlier works^10,37–39^. The modeling studies with deep neural networks^21,22,40–43^, which allow to rapidly estimate the turbulent diusivities from the several known physical parameters, recently become more active.

In our previous work^44^ and a related work^45^ by means of the nonlinear gyrokinetic turbulence simulations, a novel regression model that describes a nonlinear functional relation(NFR) between the turbulent ion heat diusivity, the turbulence intensity, and the zonal-ow intensity has been proposed. As will briefly be reviewed in Section “Construction of nonlinear functional relation”, the NFR is identified by utilizing mathematical optimization techniques to find the optimal parameter sets as the extremum point in the multi–dimensional solution space. The NFR successfully reproduced the turbulent diffusivity in the nonlinear gyrokinetic simulations for a wide range of physical parameters and the radial domains, including near– and far–marginal ITG stability^44^. Then, the regression error of $\sim 7.88$% has been observed to be much lower than that in the earlier works^30–34^. It should be noted that the NFR is a regression model which still needs the nonlinear simulation data of the turbulence and zonal–flow intensities. Further modeling is necessary to reduce the NFR to the turbulent transport model that can be applied to the integrated transport simulations.

To this end, we propose an extended modeling to realize a novel simplified turbulent transport model. Here the turbulence intensity and the zonal-ow intensity are represented by nonlinear functions of the instability growth rate and the zonal-ow decay time obtained from the “linear” gyrokinetic simulations which are more than 100 times faster than the typical nonlinear simulations. Particularly, the turbulence suppression by the zonal–flows and its temperature-gradient dependence, which have been ignored in earlier works^30–34^, are incorporated. The optimal parameters in the nonlinear model functions are determined by using the mathematical optimization technique which is further extended from that used in the previous work on the NFR^44^.

The rest of the paper is organized as follows. The gyrokinetic model and the nonlinear simulation dataset for a tokamak ITG turbulence are shown in Section “Gyrokinetic simulation model and nonlinear simulation dataset”. Then, the construction of NFR by using the mathematical optimization technique is demonstrated in Section “Construction of nonlinear functional relation”. Section Further simplication to transport model” discusses the further modeling from the NFR to the simplified transport model, including the verification of the regression and extrapolation errors. The summary is given in Section “Summary”.

## Gyrokinetic simulation model and nonlinear simulation dataset

The gyrokinetic turbulence simulation model and numerical results are shown in this section. Datasets of the fluctuation intensity for the turbulence and zonal–flow potential components, and the turbulent heat diffusivity are used in constructing the nonlinear functional relation(NFR) in Section “Construction of nonlinear functional relation”.

The ITG driven turbulence simulations with a tokamak geometry are carried out by using a gyrokinetic Vlasov simulation code GKV^46^ which is represented in the local uxtube coordinates. The electrostatic limit with the adiabatic electron response is assumed because the modeling incorporating the turbulence nonlinearity and the zonal-ow eects, which has been revealed by the nonlinear interaction analysis^47^, is of particular focus in this study. The gyrokinetic-Poisson equations in Fourier wavenumber representation are summarized as follows:

1 $$\begin{aligned} \left[ \frac{\partial }{\partial t} +v_{\parallel }\nabla _{\parallel } + i \omega _{\textrm{Ds}} - \left( \frac{e_{\textrm{s}}\mu }{m_{\textrm{i}}}\nabla _\parallel B\right) \frac{\partial }{\partial v_\parallel }\right] \delta f_{\textrm{s}\varvec{k}_\perp }- & {} \frac{1}{B}\sum _{\varvec{k'_\perp +k''_\perp =k_\perp }}\varvec{b}\cdot (\varvec{k'_\perp \times k''_\perp })J_0(k'_{\perp }\rho _{\textrm{s}})\delta {\phi }_{\varvec{k'}_\perp }\delta f_{\textrm{s}\varvec{k''}_\perp }\nonumber \\= & {} \frac{e_{\textrm{s}}F_{\textrm{Ms}}}{T_{\textrm{s}}}(i\omega _{*T\textrm{s}}+i\omega _{\textrm{Ds}}-v_\parallel \nabla _{\parallel })J_0(k_{\perp }\rho _{\textrm{s}})\delta {\phi }_{\varvec{k}_\perp }+C_{\textrm{s}}, \end{aligned}$$

2 $$\begin{aligned} \bigg [k_{\perp }^2+\frac{1}{\varepsilon _0}\sum _{\textrm{s}}\frac{e_{\textrm{s}}^2n_{\textrm{s}}}{T_{\textrm{s}}}(1-\Gamma _{0{\textrm{s}\varvec{k}_\perp }})\bigg ]\delta {\phi }_{\varvec{k}_\perp }= & {} \frac{1}{\varepsilon _0}\sum _{\textrm{s}} e_{\textrm{s}}\int d\varvec{v} \,J_0(k_{\perp }\rho _{\textrm{s}}) \delta f_{\textrm{s}\varvec{k}_\perp }, \end{aligned}$$

where $\delta f_{\textrm{s}\varvec{k}_\perp } = \delta f_{\textrm{s}\varvec{k}_\perp } (z, v_{\parallel }, \mu , t)$ means the perturbed gyrocenter distribution function for the particle species “$\textrm{s}$”. Here, $\varvec{k}_\perp = (k_x, k_y)$, $\varvec{b}$, *B*, $\delta \phi _{\varvec{k}_\perp }$, $\mu$, $e_{\textrm{s}}$, $m_{\textrm{s}}$, and $T_{\textrm{s}}$ correspond to the perpendicular wavenumber vector, the unit vector parallel to the field line, the magnetic field strength, the electrostatic potential fluctuation, the magnetic moment, the electric charge, the particle mass, and the temperature for each particle species, respectively. The drift frequency, the diamagnetic frequency, and the gyroradius are $\omega _{\textrm{Ds}}$, $\omega _{*T{\textrm{s}}}$, and $\rho _{\textrm{s}}$, respectively. The finite gyroradius effects are denoted by the 0th-order Bessel function $J_0$ and $\Gamma _0 = e^{-b}I_0(b)$ with $b=(k_\perp \rho _{\textrm{ti}})^2$, where the 0th-order modified Bessel function, the ion thermal speed, and the ion thermal gyroradius are $I_0$, $v_{\mathrm {{ti}}}=\sqrt{T_{\textrm{i}}/m_{\textrm{i}}}$, and $\rho _{\mathrm {{ti}}}=m_{\textrm{i}}v_{\textrm{ti}}/e_{\textrm{i}}B$, respectively. The maxwellian distribution and the collision operator are corresponding to $F_{\textrm{M}}$ and $C_{\textrm{s}}$, respectively. Note that the present simulation model Eq. (1) ignores the background profile variations, the parallel nonlinearity, and the coupling of $\varvec{E}\times \varvec{B}$ and magnetic drifts in the context of delta–f formalism.

The physical and numerical parameters used in the nonlinear simulation are summarized in table 1. The simulations are performed for the various normalized radial positions $\rho =r/a$ and for the normalized logarithmic temperature gradient $R/L_T = R(-\partial \textrm{ln}T_{\textrm{s}}/\partial r)$, where *r*, *a*, and *R* correspond to the radial position, the minor radius, and the major radius of the plasma, respectively. The safety factor and the magnetic shear are denoted by *q* and $\hat{s}$. The logarithmic density gradient $R/L_{\textrm{n}}$ and temperature ratio between electron and ion $T_{\textrm{e}}/T_{\textrm{i}}$ are fixed to 2.2 and 1.0, respectively. The phase-space grid number and minimum wavenumber are denoted by $(n_{k_x}, n_{k_y}, n_z, n_{v_\parallel }, n_\mu )$ and $(\Delta k_x, \Delta k_y)$, respectively, where $\mu \equiv m_{\textrm{s}}v_\perp ^2/2B$. The velocity-space grid number is fixed as $(n_{v_\parallel }, n_\mu )=(48,12)$, and the field–line grid number is $n_z=64$. The velocity domain of $0\le v_{\perp }\le 4v_{\textrm{ti}}$, and $-4v_{\textrm{ti}}\le v_{\parallel }\le 4v_{\textrm{ti}}$ are considered. Here, 44 simulations for the weakly collisional ITG driven turbulence are performed.

**Table 1:** Physical and numerical parameters used in the simulations.

Parameter	Value and range			
Radial position $\rho$	0.25	0.5	0.6	0.75
Safety factor *q*	1.00	1.41	1.70	2.39
Magnetic shear $\hat{s}$	0.231	0.885	1.23	1.84
Temperature gradient $R/L_T$	5.1 to 12	4.7 to 12	4.25 to 12	4.2 to 9
Density gradient $R/L_n$	2.2	2.2	2.2	2.2
Collisionality $\nu _{ii}^*$	0.056	0.056	0.056	0.056
Minimum wavenumber $(\Delta k_x, \Delta k_y)$	(0.054, 0.075)	(0.069,0.075)	(0.073,0.075)	(0.072,0.075)
$\varvec{k}$–space grid number $(n_{k_x}, n_{k_y})$	($\pm 24,20$)	($\pm 64,20$)	($\pm 96,20$)	($\pm 128,20$)
Field line grid number $n_z$	64	64	64	64
Velocity–space grid number $(n_{v_\parallel }, n_\mu )$	($\pm 24$,12)	($\pm 24$,12)	($\pm 24$,12)	($\pm 24$,12)

**Figure 1:**
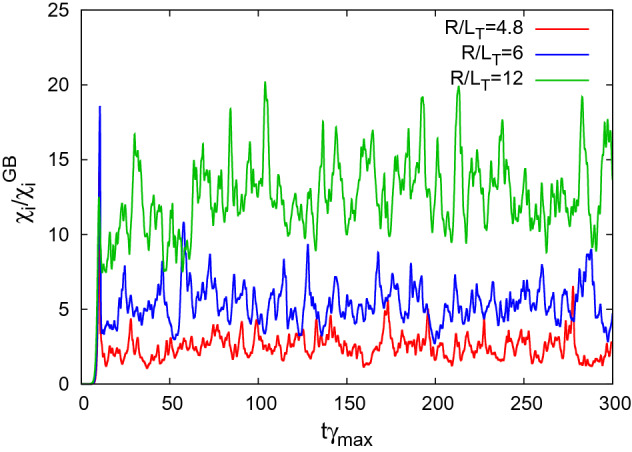
Time evolutions of turbulent heat diffusivity $\chi _{\textrm{i}}/\chi _{\textrm{i}}^{\textrm{GB}}$ at $\rho =0.5$ for several temperature gradients, obtained from the nonlinear gyrokinetic simulations.

The turbulent transport level(heat diffusivity normalized by the gyro-Bohm unit) ${\chi }_{\textrm{i}}/\chi _{\textrm{i}}^{\textrm{GB}}$, the turbulence intensity $\mathcal{T}$, and the zonal–flow intensity $\mathcal{Z}$ are obtained from the nonlinear gyrokinetic simulation, where $\mathcal{T}$ and $\mathcal{Z}$ are defined as

3 $$\begin{aligned} \mathcal{T}= & {} \frac{1}{2}\sum _{k_x,k_y\ne 0}\left\langle | \delta \phi _{k_x,k_y}|^2 \right\rangle , \end{aligned}$$

4 $$\begin{aligned} \mathcal{Z}= & {} \frac{1}{2}\sum _{k_x}\left\langle | \delta \phi _{k_x,k_y=0}|^2 \right\rangle , \end{aligned}$$

respectively. Here, the symbol $\langle \cdots \rangle$ means the flux–surface average. $\mathcal{T}$ and $\mathcal{Z}$ are obtained as the outputs in the nonlinear simulation, so that they depend not only on the profile gradients, but also on the magnetic eld structure and the collisionality. Figure 1 shows the time evolution of the heat diffusivity at $\rho =0.5$. Note that the time in the horizontal axis is normalized by the maximum growth rate $\gamma _{\textrm{max}}$ to unify the time scale of turbulent fluctuations for the cases with different growth rates of the ITG instability. In this paper, time averaged values of $\bar{\chi }_{\textrm{i}}/\chi _{\textrm{i}}^{\textrm{GB}}$, $\bar{\mathcal{T}}$, and $\bar{\mathcal{Z}}$ are considered, where the time window of $100\le t\gamma _{\textrm{max}}\le 300$ is used.

**Figure 2:**
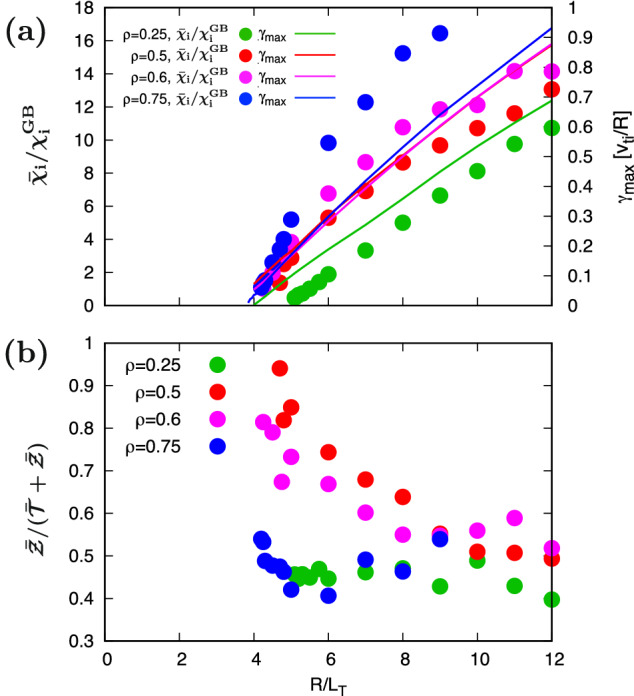
Temperature gradient dependence of (**a**) turbulent heat diffusivity $\bar{\chi }_{\textrm{i}}/\chi _{\textrm{i}}^{\textrm{GB}}$ (symbols) and the maximum instability growth rate $\gamma _{\textrm{max}}$ (lines), and (**b**) ratio of the zonal–flow intensity to the total turbulence intensity $\bar{\mathcal{Z}}/(\bar{\mathcal{T}}+\bar{\mathcal{Z}})$.

The $R/L_T$–dependence of $\bar{\chi }_{\textrm{i}}/\chi _{\textrm{i}}^{\textrm{GB}}$ and the maximum ITG instability growth rate $\gamma _{\textrm{max}}$ are summarized in Fig. 2(a). One can see the so-called Dimits-shift^48^, which indicates a slight difference between the critical gradient of the ITG instability and the effective gradient driving the thermal transport. Figure 2(b) shows the ratio of the zonal–flow potential intensity to the total fluctuation intensity $\bar{\mathcal{Z}} /(\bar{\mathcal{T}} + \bar{\mathcal{Z}})$ as a function of $R/L_T$. A relatively larger zonal–flow ratio $\bar{\mathcal{Z}}/(\bar{\mathcal{T}} +\bar{\mathcal{Z}})$ is observed in the region near the critical gradient. For the inner–core and outer–core regions with slightly different *q* and $\hat{s}$, a moderate $R/L_T$–dependence is observed. The weaker $R/L_T$–dependence appears in the region with larger temperature gradient of $R/L_T \ge 6$ that means the far–marginal stability.

## Construction of nonlinear functional relation

Utilizing the dataset shown in the previous section, one can construct the nonlinear functional relation(NFR) that approximately reproduces the nonlinear simulation results, where the dataset for $\rho =0.6$ is added in the present analysis. The NFR was dened as follows:

5 $$\begin{aligned} \frac{\bar{\chi }_{\textrm{i}}}{\chi _{\textrm{i}}^{\textrm{GB}}}\sim F^{\textrm{NFR}}(\bar{\mathcal{T}},\bar{\mathcal{Z}})=\frac{C_1\bar{\mathcal{T}}^{\alpha }}{1+C_2(\bar{\mathcal{Z}}^{\xi }/\bar{\mathcal{T}})^\beta }. \end{aligned}$$

Here, $(C_1,C_2,\alpha , \beta , \xi )$ is the regression parameters to be determined. The transport suppression by the zonal–flows is represented by $\bar{\mathcal{Z}}^{\xi }/\bar{\mathcal{T}}$ in the denominator. It is noted that the functional form of $F^{\textrm{NFR}}$ is not unique, but is chosen to satisfy phenomenological requirements among $\bar{\mathcal{T}}$, $\bar{\mathcal{Z}}$, $\bar{\chi }_{\textrm{i}}/{\chi }_{\textrm{i}}^{\textrm{GB}}$ as follows;

6 $$\begin{aligned} F^{\textrm{NFR}}(\mathcal{{\bar{T},\bar{Z}}})\ge & {} 0, \nonumber \\ \lim _{\bar{\mathcal{T}}\rightarrow 0}\,F^{\textrm{NFR}}(\mathcal{{\bar{T},\bar{Z}}})= & {} 0, \nonumber \\ \lim _{\bar{\mathcal{T}}\rightarrow \infty }\,F^{\textrm{NFR}}(\mathcal{{\bar{T},\bar{Z}}})= & {} C_1\bar{\mathcal{T}}^{\alpha }, \\ \lim _{\bar{\mathcal{Z}} \rightarrow 0}\, F^{\textrm{NFR}}(\mathcal{{\bar{T},\bar{Z}}})= & {} C_1\bar{\mathcal{T}}^{\alpha }, \nonumber \\ \lim _{\bar{\mathcal{Z}}\rightarrow \infty }\,F^{\textrm{NFR}}(\mathcal{{\bar{T},\bar{Z}}})= & {} 0 \nonumber . \end{aligned}$$

These requirements mean that the turbulent transport coefficient should be positive, that the transport does not occur in the limits of $\bar{\mathcal{T}}\rightarrow 0$ and $\bar{\mathcal{Z}}\rightarrow \infty$, and that the transport is asymptotically determined only by $\bar{\mathcal{T}}$ in the limits of $\bar{\mathcal{T}}\rightarrow \infty$ and $\bar{\mathcal{Z}}\rightarrow 0$.

Mathematical optimization techniques are applied to determine the regression parameters $(C_1,C_2,\alpha , \beta , \xi )$ in Eq. (5). The extremum point to minimize the regression error (or the objective function in the context of mathematical optimization) $\sigma _{\textrm{NFR}}$ is searched in 5-dimensional solution space, where $\sigma _{\textrm{NFR}}$ is dened by the root–square–mean deviation as follows:

7 $$\begin{aligned} {\sigma _{\textrm{NFR}}}=\sqrt{\frac{1}{n}\sum _{j=1}^n\left( \frac{F^{\textrm{NFR}}(\bar{\mathcal{T}}_j, \bar{\mathcal{Z}}_j)}{\bar{\chi }_{\textrm{i},j}/\chi _{\textrm{i}}^{\textrm{GB}}}-1\right) ^2}. \end{aligned}$$

Here, *j* and *n* mean the data index and the total number of data, respectively. Since the regression error $\sigma _{\textrm{NFR}}$ is a multi-modal function in $(C_1, C_2, \alpha , \beta , \xi )$–space, one must care about the possibility of the several local minima. Therefore, the search for a single initial value is not sucient, and it is effective to cover as wide a range of initial conditions as possible.

According to our previous work^44^, a hybrid optimization algorithm with the Hesseian- and gradient-based scheme, which is similar to Levenberg-Marquardt method^49,50^, is applied. The numerical scan is performed in the ten combinations of the two-dimensional subspace given by the combinations of selected two initial values from the five initial values, where the other three unselected values are fixed. This enables us to reduce the numerical costs for the original five-dimensional space, keeping the characteristic of global search. In the present hybrid algorithm, the Newton method which has fast convergence at near local minima is used when the Hessian matrix is the positive denite. Otherwise, the gradient descent method that ensures global convergence is applied, so that the hybrid algorithm holds both global convergence and locally fast convergence. Furthermore, Monte–Carlo–like scheme is utilized to verify the robustness of obtained optimal parameters, where randomly given points near the optimal solution are re–evaluated as the initial condition to confirm the attractiveness (i.e., stability) toward the optimal solution.

**Figure 3:**
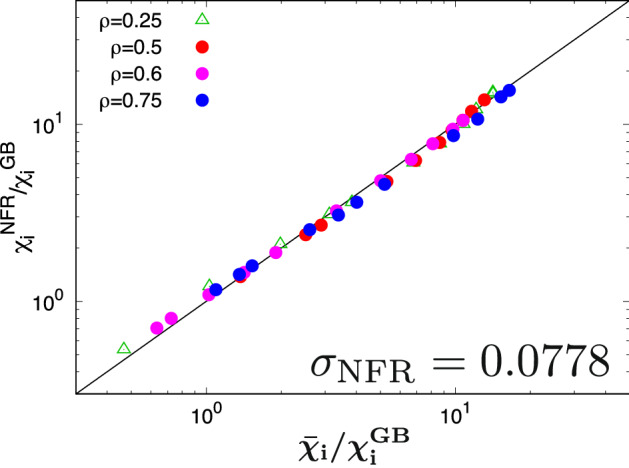
Comparison of the turbulent heat diffusivities between the nonlinear gyrokinetic simulation results $\bar{\chi }_{\textrm{i}}/\chi _{\textrm{i}}^{\textrm{GB}}$ and the NFR–estimated $\chi _{\textrm{i}}^{\textrm{NFR}}/{\chi _{\textrm{i}}^{\textrm{GB}}}$, where the datasets of $\rho =0.5$, 0.6, 0.75 are used in the regression.

In this work, the regression with Eqs. (5) and (7) is performed by using the dataset of $(\bar{\mathcal{T}},\bar{\mathcal{Z}},\bar{\chi }_{\textrm{i}}/\chi _{\textrm{i}}^{\textrm{GB}})$ for the various temperature gradient parameters, where three radial positions of $\rho = 0.5$, 0.6, and 0.75 are considered. The dataset of $\rho = 0.25$ is used in the verication of extrapolation. We found that the optimal regression parameters and the regression error are $(C_1, C_2, \alpha , \beta , \xi ) = (0.500, 1.06, 0.595, 0.854, 0.201)$ and $\sigma _{\textrm{NFR}} = 0.0778$, respectively. These regression parameters are consistent with our previous work^44^ in spite of using the slightly different numerical and physical parameters. Note that the regression error is $2\sim 4$ times smaller than that in earlier works [$\sigma =0.159$(Ref.^30^), 0.12(Ref.^31^), 0.15(Ref.^33^), 0.30(Ref.^34^)]. The comparison between the present NFR and the nonlinear gyrokinetic simulation results is shown in Fig. 3, where the turbulent heat diffusivity is well reproduced ($\rho =0.5$, 0.6, 0.75) and extrapolated ($\rho =0.25$) for a wide parameter range corresponding to $0.4<\bar{\chi }_{\textrm{i}}/\chi _{\textrm{i}}^{\textrm{GB}}<17$.

## Further simplication to transport model

In the previous section, a good estimation accuracy of the NFR was demonstrated. Here, the additional modeling for the $\bar{\mathcal{T}}$ and $\bar{\mathcal{Z}}$ appearing in Eq. (5) is proposed for constructing a novel turbulence transport model.

**Figure 4:**
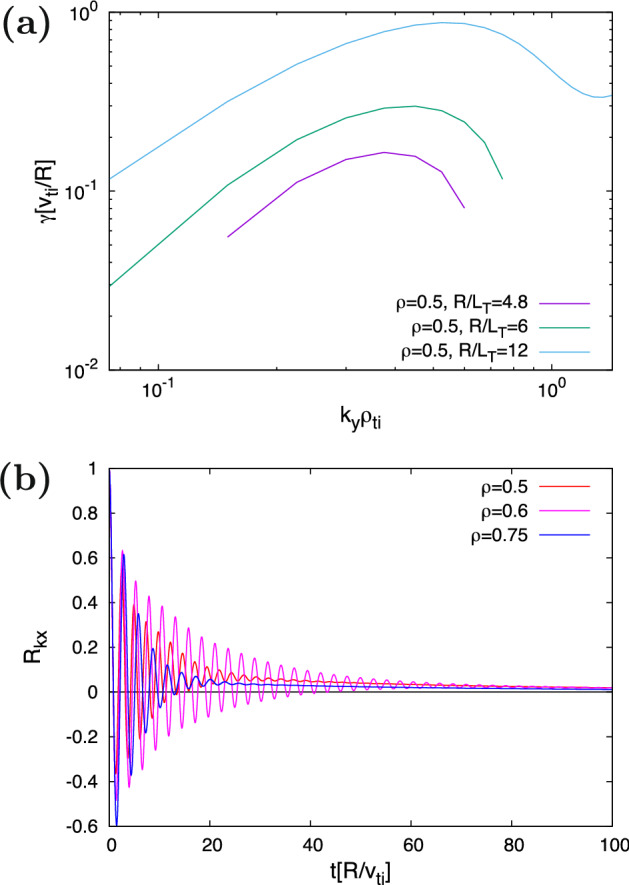
(**a**) The wavenumber spectra of the ITG instability growth rate $\gamma$ at the $\rho =0.5$ and (**b**) zonal–flow response function ${\mathcal{R}_{kx}(t)}$ at each radial positions, calculated by the linear gyrokinetic simulations.

Following earlier works^30–34^, $\bar{\mathcal{T}}$ and $\bar{\mathcal{Z}}$ are approximated by the quantities obtained only by the “linear” simulations, such as the growth rate $\gamma _{k_x,k_y}$ of the ITG instability and the zonal-ow response function ${\mathcal{R}_{k_x}(t)}\equiv { \langle \delta \phi _{k_x,k_y=0}(t)\rangle }/{\langle \delta \phi _{k_x,k_y=0}(0)\rangle }$. The wavenumber spectrum of $\gamma _{k_x,k_y}$ for several $R/L_T$ and the zonal-ow response function ${\mathcal{R}_{k_x}(t)}$ for several radial positions are shown in Fig. 4 (a,b), respectively. For the ITG instability, only the most unstable modes with $k_x=0$ are considered. For the zonal–flow response, $k_x$ is fixed to $\sim 0.1$ that is a typical radial scale of zonal–flows in the ITG turbulence. Note also that the zonal-ow response function does not depend on the temperature and density gradients, but depends on the magnetic congurations or the radial positions. On the other hand, the nonlinear simulation results[see Fig. 2 b] indicate the strong gradient dependence. Thus, one need a special care of incorporating the gradient dependence to the modeling of the zonal–flow response, but earlier works^30–34^ ignored the gradient dependence.

**Figure 5:**
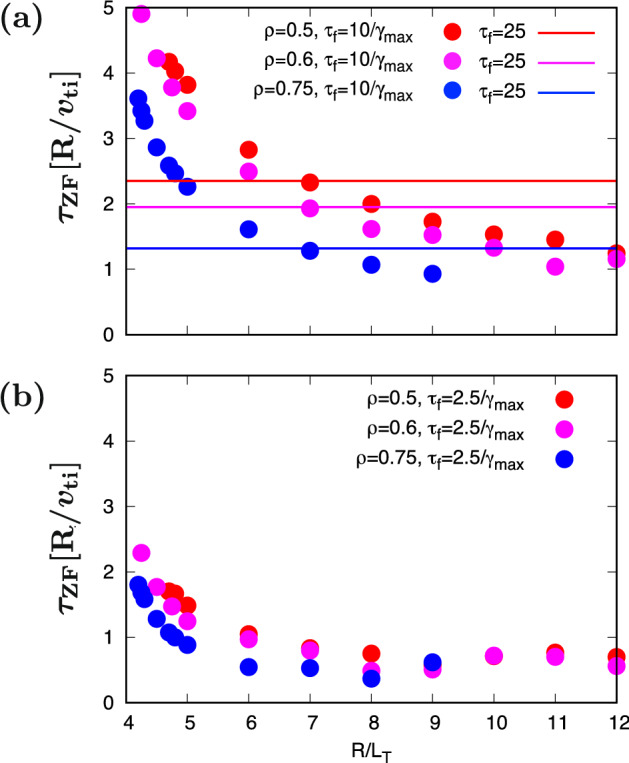
Temperature gradient dependence of zonal–flow decay time $\tau _{\textrm{ZF}}\equiv \int _{0}^{\tau _{\textrm{f}}}dt{\mathcal{R}_{kx}(t)}$(symbols), where $\tau _{\textrm{f}}\equiv C/\gamma _{\textrm{max}}$. (**a**) $C=10$ and (**b**)$C=2.5$. The horizontal lines in (**a**) indicate the gradient–independent $\tau _{{ZF}}$, assumed in earlier works^30–34^.

In order to approximate $\bar{\mathcal{T}}$ and $\bar{\mathcal{Z}}$ by the quantities obtained from the linear calculations, the mixing–length diffusivity^51^ $\mathcal{L}$ and the zonal-ow decay time $\tau _{\textrm{ZF}}$ are introduced:

8 $$\begin{aligned} \mathcal{L}\equiv & {} \sum _{k_y}\frac{\gamma _{{k_y}}}{{k_y}^2}, \end{aligned}$$

9 $$\begin{aligned} \tau _{\textrm{ZF}}\equiv & {} \int ^{{\tau _{\textrm{f}}(\gamma _{\textrm{max}})}}_0 dt\ {\mathcal{R}_{k_x}(t)}, \end{aligned}$$

where the upper limit of the integral interval $\tau _{\textrm{f}}(\gamma _{\textrm{max}})$ , which is a function of the maximum growth–rate $\gamma _{\textrm{max}}$, is given by a typical time scale of the turbulence growth and the correlation, i.e., $\tau _{\textrm{f}}=C/\gamma _{\textrm{max}}$ with a constant *C*. $\mathcal{R}_{k_x}(t)$ means the time–dependent zonal–flow response function, corresponding to the Rosenbluth-Hinton kernel^52^. It is noted that, in contrast to ${\mathcal{R}_{k_x}(t)}$, the zonal–flow decay time $\tau _{\textrm{ZF}}$ is gradient–dependent through the temperature and density gradient dependence in the maximum ITG growth rate $\gamma _{\textrm{max}}$. Indeed, the $R/L_T$–dependence found in the nonlinear simulations [Fig. 2 b] is qualitatively reproduced, as shown in Fig. 5 (a), where $C=10$. For comparison, the gradient-independent $\tau _{\textrm{ZF}}$ used in the earlier works is also displayed by the horizontal lines in the figure. Although $\tau _{\textrm{f}}$ includes a control parameter *C*, one can see that, as shown in Fig. 5(b), the qualitative characteristic of $R/L_T$–dependence still holds even for $C=2.5$. In the followings, $C=10$ is selected in view of the total performance in the regression and extrapolation.

### Turbulence intensity modeling

**Figure 6:**
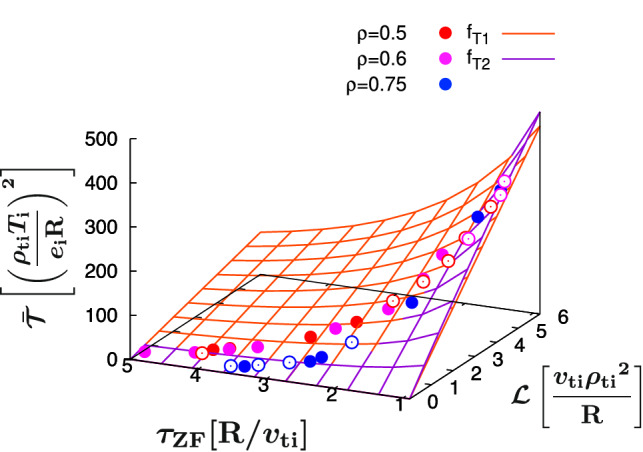
Distribution of $\bar{\mathcal{T}}$ in $\mathcal{L}$–$\tau _{\textrm{ZF}}$ space, and overplot of the surfaces [Eqs. (10 and 11)] to reproduce $\bar{\mathcal{T}}$. The open symbols denote the data points located behind the surfaces.

A model for the turbulence intensity $\bar{\mathcal{T}}$ is discussed in this subsection. It is stressed that the modeling only by $\mathcal{L}$ (or similarly by $\gamma _{k_y}$) is not appropriate particularly for the cases in the near–marginal stability, because the turbulence intensity $\bar{\mathcal{T}}$ is influenced by the suppression effects of zonal–flows. Indeed, the distribution of $\bar{\mathcal{T}}$ in $\mathcal{L}$–$\tau _{\textrm{ZF}}$ space shown by the symbols in Fig. 6 indicates a strong dependence on both $\mathcal{L}$ and $\tau _{\textrm{ZF}}$. $\bar{\mathcal{T}}$ is a decaying function of $\tau _{\textrm{ZF}}$, indicating a steeper gradient for $\tau _{\textrm{ZF}} < 2$. For convenience, the low–$\tau _{\textrm{ZF}}$ and high–$\tau _{\textrm{ZF}}$ regions are dened by $\tau _{\textrm{ZF}} < 2$ and $\tau _{\textrm{ZF}} \ge 2$, respectively. Then, the model function for $\bar{\mathcal{T}}$ has to reproduce such tendency. We consider two kinds of the model function in the similar manner for the NFR that satisfies the phenomenological requirements as discussed in the previous section. The model functions $f_{\mathcal{T}1}$ and $f_{\mathcal{T}2}$ are dened as:

10 $$\begin{aligned} \bar{\mathcal{T}}\sim & {} f_{\mathcal{T}1}\left( \mathcal{L},\tau _{\textrm{ZF}}\right) =\frac{C_{\mathcal{T}1}\mathcal{L}^{\alpha _{\mathcal{T}}}}{1+C_{\mathcal{T}2}{{\tau _{\textrm{ZF}}}^{\beta _{\mathcal{T}}}}}, \end{aligned}$$

11 $$\begin{aligned} \bar{\mathcal{T}}\sim & {} f_{\mathcal{T}2}\left( \mathcal{L},\tau _{\textrm{ZF}}\right) ={\frac{C^{\prime }_{\mathcal{T}}\mathcal{L}^{\alpha ^{\prime }_{\mathcal{T}}}}{\textrm{exp}(\beta ^{\prime }_{\mathcal{T}1}{\tau _{\textrm{ZF}}}^{\beta ^{\prime }_{\mathcal{T}2}})}}, \end{aligned}$$

where $(C_{\mathcal{T}1}, C_{\mathcal{T}2},\alpha _{\mathcal{T}}, \beta _{\mathcal{T}})$ and $(C^{\prime }_{\mathcal{T}}, \alpha ^{\prime }_{\mathcal{T}}, \beta ^{\prime }_{\mathcal{T}1}, \beta ^{\prime }_{\mathcal{T}2})$ are regression parameters to be determined. The zonal-ow eects corresponding to the strong decay of $\bar{\mathcal{T}}$ in the low–$\tau _{\textrm{ZF}}$ region are treated in the form of a rational or an exponential function in $f_{\mathcal{T}1}$ and $f_{\mathcal{T}2}$, respectively.

The optimal regression parameters are identified by the mathematical optimization in similar way described in Section “Construction of nonlinear functional relation”, where the regression error $\sigma _\mathcal{T}$ is dened as follows:

12 $$\begin{aligned} {\sigma _\mathcal{T}}^2=\frac{1}{n}\sum _j^n \left( \frac{f_{\mathcal{T}}\left( \mathcal{L}_j,\tau _{\textrm{ZF},j}\right) }{\bar{\mathcal{T}}_j}-1\right) ^2, \end{aligned}$$

for $f_\mathcal{T}=f_{\mathcal{T}1}$ or $f_\mathcal{T}=f_{\mathcal{T}2}$. The optimal regression parameters are identified as $(C_{\mathcal{T}1},C_{\mathcal{T}2},\alpha _{\mathcal{T}}, \beta _{\mathcal{T}})=(100, 0.472, 0.996, 1.50)$ and $(C^{\prime }_{\mathcal{T}}, \alpha ^{\prime }_{\mathcal{T}}, \beta ^{\prime }_{\mathcal{T}1}, \beta ^{\prime }_{\mathcal{T}2})=(520, 0.970, 1.93, 0.365)$ for $f_{\mathcal{T}1}$ and $f_{\mathcal{T}2}$, respectively, where the regressions error are $\sigma _\mathcal{T} = 0.181(f_{\mathcal{T}1})$, and $\sigma _\mathcal{T} = 0.179(f_{\mathcal{T}2})$. As shown by the surfaces in Fig. 6, either functional form of $f_{\mathcal{T}1}$ or $f_{\mathcal{T}2}$ reasonably reproduces the nonlinear simulation dataset $\bar{\mathcal{T}}$. Equation (11), which has a slightly smaller error, is considered in the followings.

### Zonal–flow intensity modeling

In this subsection, the modeling for the zonal-ow suppression effect $\bar{\mathcal{Z}}^{\xi }/\bar{\mathcal{T}}$ appearing in the NFR [Eq. 5] is presented. From the view point of the similarity to the zonal–flow response function ${\mathcal{R}_{k_x}(t)}$, the relative zonal–flow intensity $\bar{\mathcal{Z}}/\bar{\mathcal{T}}$ [or equivalently $\bar{\mathcal{Z}}/(\bar{\mathcal{T}}+\bar{\mathcal{Z}})$ in Fig. 2(b)] is modeled here. Since the parameter $\xi$ is an optimal parameter in the determination of the NFR, the value may change for significantly different magnetic configuration such as stellarators.

**Figure 7:**
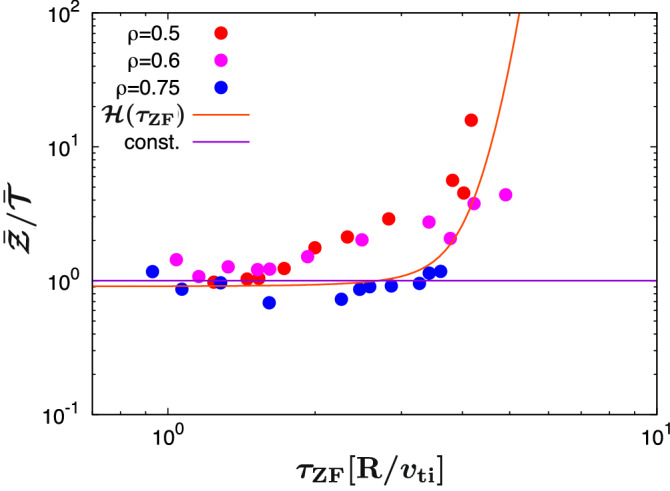
$\tau _{\textrm{ZF}}$–dependence of $\bar{\mathcal{Z}}/ \bar{\mathcal{T}}$. The model function $\mathcal{H}$ is shown by the curve. The horizontal line means the constant value of 1.

Thus, the model function for the zonal–flow suppression effect should not include $\xi$. To treat $\xi$ separately, zonal–flow suppression term in NFR is rewritten as;

13 $$\begin{aligned} \frac{\bar{\mathcal{Z}}^\xi }{\bar{\mathcal{T}}}= & {} {\bar{\mathcal{T}}}^{(\xi -1)}\left( \frac{\bar{\mathcal{Z}}}{\bar{\mathcal{T}}}\right) ^\xi \nonumber \\\sim & {} \left[ {f_{\mathcal{T}2}}(\mathcal{L},\tau _{\textrm{ZF}})\right] ^{(\xi -1)}\left[ \mathcal{H}(\tau _{\textrm{ZF}})\right] ^\xi , \end{aligned}$$

where Eq. (11) is used for $\bar{\mathcal{T}}^{(\xi -1)}$. Also, $\mathcal{H}(\tau _{\textrm{ZF}})$ is an additional model function to approximate $\bar{\mathcal{Z}}/\bar{\mathcal{T}}$ by $\tau _{\textrm{ZF}}$. Figure 7 shows the relation between $\tau _{\textrm{ZF}}$ and $\bar{\mathcal{Z}}/\bar{\mathcal{T}}$. It shows that $\bar{\mathcal{Z}}/\bar{\mathcal{T}}\sim \mathrm {const.}$ in the region of $\tau _{\textrm{ZF}}<2$, and increases with $\tau _{\textrm{ZF}}$ in the region of $\tau _{\textrm{ZF}}\ge 2$. From this observation, the functional form of $\mathcal{H}$ is dened as follows:

14 $$\begin{aligned} \frac{\bar{\mathcal{Z}}}{\bar{\mathcal{T}}}\sim \mathcal{H}(\tau _{\textrm{ZF}};C_{\mathcal{Z}1},C_{\mathcal{Z}2},\alpha _\mathcal{Z},\beta _\mathcal{Z})=C_{\mathcal{Z}1}+C_{\mathcal{Z}2}\textrm{exp}\left( \alpha _\mathcal{Z}{\tau _{\textrm{ZF}}}^{\beta _\mathcal{Z}}\right) . \end{aligned}$$

In the similar manner for $F^{\textrm{NFR}}$ and $\bar{\mathcal{T}}$, the optimal regression parameter are identified as $(C_{\mathcal{Z}1},C_{\mathcal{Z}2},\alpha _\mathcal{Z},{\beta _\mathcal{Z}})=(0.883, 0.0236, 0.140, 2.47)$ with the error $\sigma _\mathcal{Z}=0.354$. A relatively larger error compared to the modeling for $\bar{\mathcal{T}}$ is associated with scattered distribution of $\bar{\mathcal{Z}}/\bar{\mathcal{T}}$ for $\tau _{\textrm{ZF}}\ge 2$ in Fig. 7. On the other hand, a nearly-constant characteristic for the smaller $\tau _{\textrm{ZF}}$ is well described in Eq. (14).

### Construction of simplified transport model and its verification

Finally, the above modeling is combined to the NFR in this subsection. Substituting the model functions Eqs. (11 and 14) into Eq. (3), a novel transport model for the ITG driven turbulent heat diffusivity is expressed as follows:

15 $$\frac{\bar{\chi }_{\textrm{i}}}{\chi _{\textrm{i}}^{\textrm{GB}}}\sim \frac{{\chi }_{\textrm{i}}^{\textrm{model}}}{\chi _{\textrm{i}}^{\textrm{GB}}}\left( {\mathcal{L}},\tau _{\textrm{ZF}}\right) =\frac{\Theta _{1}{\mathcal{L}}^{\Theta _{2}}{\textrm{exp}}\left( \Theta_{3}{\tau _{\textrm{ZF}}}^{\Theta_{4}}\right) }{1+\Theta_{5}{\mathcal{L}}^{\Theta_{6}}{\textrm{exp}}\left( \Theta_{7}{\tau_{\textrm{ZF}}}^{\Theta_{4}}\right) \left[ {\mathcal{H}}(\tau _{\textrm{ZF}})\right] ^{\Theta_{8}}}.$$

Here, $\{\Theta _{\textrm{i}}\}$ for $1\le \textrm{i}\le 8$ are the parameters, which were already determined by the combinations of the regression parameters summarized in table 2. The model function $\mathcal{H}(\tau _{\textrm{ZF}})$ with $(C_{\mathcal{Z}1},C_{\mathcal{Z}2},\alpha _\mathcal{Z},\beta _\mathcal{Z})$ is given by Eq. (14).

**Table 2:** Parameters in the simplified transport model.

Parameters	Expression	Value
$\Theta _1$	$C_1{C_\mathcal{T}^{\prime }}^\alpha$	20.7
$\Theta _2$	$\alpha \alpha _\mathcal{T}^\prime$	0.577
$\Theta _3$	$-\alpha \beta _{\mathcal{T}1}^\prime$	–1.15
$\Theta _4$	$\beta ^{\prime }_{\mathcal{T}2}$	0.365
$\Theta _5$	$C_2{C_\mathcal{T}^{\prime }}^{\beta (\xi -1)}$	0.0127
$\Theta _6$	$\beta \alpha _\mathcal{T}^\prime (\xi -1)$	–0.662
$\Theta _7$	$-\beta \beta _{\mathcal{T}1}^\prime (\xi -1)$	1.32
$\Theta _8$	$\beta \xi$	0.172

**Figure 8:**
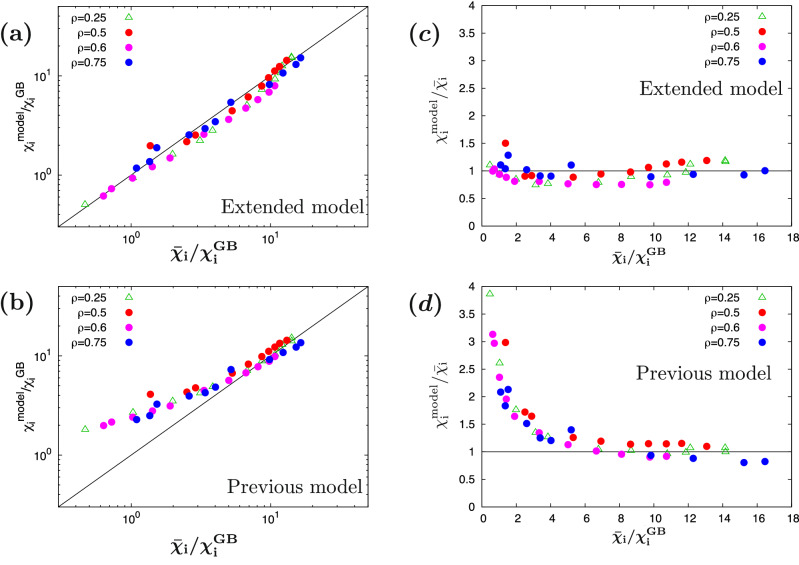
Comparison of the turbulent thermal diffusivities between the nonlinear gyrokinetic simulation results $\bar{\chi }_{\textrm{i}}/\chi _{\textrm{i}}^{\textrm{GB}}$ and simplified transport model $\chi _{\textrm{i}}^{\textrm{model}}/{\chi _{\textrm{i}}^{\textrm{GB}}}$. (**a**) The extended model in this study and (**b**) the conventional model in the earlier work^30^. The circle and triangle symbols correspond to the data for regression and extrapolation, respectively. (**c**) and (**d**) show the variances to the nonlinear gyrokinetic simulations.

The regression error of the transport model Eq. (15), $\sigma _{\textrm{model}}$ , is evaluated as $\sigma _{\textrm{model}}=0.157$ for the regression datasets($\rho = 0.5$, 0.6, and 0.75), and $\sigma _{\textrm{model}}=0.211$ for extrapolation dataset($\rho =0.25$). Figure. 8(a,b) show the comparison between the newly constructed transport model and the nonlinear gyrokinetic simulations. The result demonstrates that the present transport model shows a good estimation accuracy for the turbulent heat diffusivity $\bar{\chi}_{\textrm{i}}/\chi _{\textrm{i}}^{\textrm{GB}}$. The total regression error is similar or less compared to those in the earlier works [$\sigma =0.129$(Ref.^30^), 0.16(Ref.^31^), 0.20(Ref.^33^), 0.27(Ref.^34^)]. It is however emphasized that the present transport model is much improved to be valid for the range of $0.4<\bar{\chi }_{\textrm{i}}/\chi _{\textrm{i}}^{\textrm{GB}}<17$ corresponding to wider parameter regions including near- and far-marginal ITG stability. Indeed, as shown in Figs. 8(c,d), a significant deviation for ${\bar{\chi} _{\textrm{i}}}/\chi _{\textrm{i}}^{\textrm{GB}}\lesssim 4$ appears when the previously proposed functional form^30^ is applied to the datasets in this work, where the regression error for Figs. 8(c,d) is evaluated as $\sigma _{\textrm{model}}=0.618$. More flexible functional form with increased optimization parameters in this study enables us to improve the reproduction accuracy in the low-transport region. Note again that, the nonlinear turbulent heat diffusivity ${\bar{\chi}_{\textrm{i}}}/\chi_{\textrm{i}}^{\textrm{GB}}$ is reproduced only by linear gyrokinetic calculations with Eq. (15).

An alternative form is also derived by imposing the approximation of $\mathcal{H}(\tau _{\textrm{ZF}})\sim 1$ when one focuses on the feature of $\bar{\mathcal{Z}}/\bar{\mathcal{T}}\sim \mathrm {const.}$ in low-$\tau _{\textrm{ZF}}$ region (see Fig. 7). Such approximation leads to

16 $$\begin{aligned} \frac{\bar{\chi }_{\textrm{i}}}{\chi _{\textrm{i}}^{\textrm{GB}}}\sim \frac{{\chi }_{\textrm{i}}^{\textrm{model}}}{\chi _{\textrm{i}}^{\textrm{GB}}}\left( \mathcal{L},\tau _{\textrm{ZF}}\right) =\frac{\Theta _1\mathcal{L}^{\Theta _2}\textrm{exp}\left( \Theta _3{\tau _{\textrm{ZF}}}^{\Theta _4}\right) }{1+\Theta _5 \mathcal{L}^{\Theta _6}\textrm{exp}\left( \Theta _7{\tau _{\textrm{ZF}}}^{\Theta _4}\right) }. \end{aligned}$$

Here, the parameters are the same as Table 2. The above more simplied form with 7 parameters indicates a slight increase of the regression error $\sigma _{\textrm{model}}=0.161$ and $\sigma _{\textrm{model}} = 0.280$ for extrapolation dataset($\rho = 0.25$), but still holds a reasonable applicability to the practical analysis.

It is noted that the explicit dependence of local parameters, such as the magnetic shear, prole gradients, collisionality, no longer appears in the simplified model Eq. (15) or (16). Then, it holds a certain extent of robustness to the local parameter variations. Indeed, our previous work^44^ has confirmed a good estimation accuracy in the NFR even for the turbulence with higher collisionality^53–55^. This suggests that the present model can be applicable to the other microinstabilities driving the turbulence and zonal flows, by re-identifying the optimal parameters. In some cases, the turbulence-to-turbulence energy transfer, which is not directly modeled in the present study, may also play an important role. Even though the present model does not provide a whole description of the turbulence, it still works for estimating the mean turbulent transport heat diusivity given by the spatially-averaged low-order correlation of turbulent uctuations.

## Summary

In this paper, a novel simplified model to estimate nonlinear turbulent transport in ITG driven turbulence only by linear calculations was proposed, where the turbulent heat diffusivity was well reproduced by the NFR [Eq. 5] and the modified nonlinear function [Eqs. (15) or (16)] composed of the linear growth rate and the linear zonal–flow response.

The improved accuracy with the regression error of $\sigma _{\textrm{model}} = 0.157$ even for a wide parameter range including near– and far–marginal ITG stability was also demonstrated. Achieving such a good accuracy is attributed to an extended modeling for $\bar{\mathcal{T}}$ and $\bar{\mathcal{Z}}$ to incorporate the turbulence suppression effects and the temperature gradient dependence, as well as utilizing the hybrid mathematical optimization method with the Monte–Carlo–like scheme.

The simplied model presented here can expand the various new possibilities of the simulation studies in fusion science and plasma physics. In addition to the application to the integrated transport simulations, the flexible combination of the simplified transport model and the nonlinear gyrokinetic simulations can evaluate fast and accurately the radial profiles of the turbulent diffusivity, the turbulence intensity, and the zonal-flow intensity in the coupled global simulation approach (e.g., Ref.^56^). Since the computational cost of the simplified model is less than about 1% of the typical nonlinear simulation, it is also utilized for the study on optimizing the magnetic-field configuration, which requires a good proxy model^57,58^ for many iterative calculations to search the optimal solution.

The modeling approach here is based on the local fluxtube simulations. On the other hand, the importance of non–local and non–diusive nature in global turbulence systems has been pointed out in many earlier works. Yet fully claried, but several works showed a clear plasma–size dependence of non–local turbulence dynamics and transport characterized by the $\rho ^* = \rho _{\textrm{i}}/a$ ($\rho _{\textrm{i}}$: the ion gyroradius, *a*: the plasma minor radius). Indeed, in a suciently large plasma size with $1/\rho ^{*}\gtrsim 300$, the non–locality of ballistic heat propagations becomes moderate and the transport level is asymptotically converged to the local uxtube calculation result^37^. In this sense, although the present simplied model may not be valid for large–$\rho ^*$(small plasma) cases, it works for suciently small–$\rho ^*$(large plasma) cases that are expected in ITER– and DEMO–class plasmas.

Also, the impact of the equilibrium radial electric eld on the instability and turbulent uctuation, the phase difference eect for multiple transport channels, and the temporal dependence of zonal flows and GAMs were not considered in this study. However, such extensions can be addressed by combining the present model to related earlier works^29,33,53,59,60^. These issues contributing to increase the estimation accuracy remain as the future works.

## Data Availability

The datasets used and/or analyzed during the current study available from the corresponding author(T.N.) on reasonable request.
